# A detailed transcript-level probe annotation reveals alternative splicing based microarray platform differences

**DOI:** 10.1186/1471-2164-8-284

**Published:** 2007-08-20

**Authors:** Joseph C Lee, David Stiles, Jun Lu, Margaret C Cam

**Affiliations:** 1 Genomics Core Laboratory, National Institute of Diabetes & Digestive & Kidney Diseases, National Institutes of Health, Bethesda, MD 20892, USA; 2 Office of the Director, National Center for Biotechnology Information, National Institutes of Health, Bethesda, MD 20894, USA

## Abstract

**Background:**

Microarrays are a popular tool used in experiments to measure gene expression levels. Improving the reproducibility of microarray results produced by different chips from various manufacturers is important to create comparable and combinable experimental results. Alternative splicing has been cited as a possible cause of differences in expression measurements across platforms, though no study to this point has been conducted to show its influence in cross-platform differences.

**Results:**

Using probe sequence data, a new microarray probe/transcript annotation was created based on the AceView Aug05 release that allowed for the categorization of genes based on their expression measurements' susceptibility to alternative splicing differences across microarray platforms. Examining gene expression data from multiple platforms in light of the new categorization, genes unsusceptible to alternative splicing differences showed higher signal agreement than those genes most susceptible to alternative splicing differences. The analysis gave rise to a different probe-level visualization method that can highlight probe differences according to transcript specificity.

**Conclusion:**

The results highlight the need for detailed probe annotation at the transcriptome level. The presence of alternative splicing within a given sample can affect gene expression measurements and is a contributing factor to overall technical differences across platforms.

## Background

Microarrays have become a widely used tool to measure gene expression levels on a genome-wide basis and are available from a number of manufacturers. Each platform incorporates proprietary technology, with differences in probe design, probe bioinformatics, probe creation and deposition, reagents and protocols across platforms introducing variability into expression analysis. A large body of work has studied the reproducibility of microarray data and therefore the interchangeability of commercial platforms. A dialogue over data sets, analysis methods, and concordance measures has evolved, but no clear consensus on the level of agreement or disagreement in expression results has been reached.

It is generally understood that differences between platforms exist. The source of contention is the interpretation of the magnitude of these differences. Some conclude from the data that microarray results are sufficiently comparable across platforms [1,2]. Others caution that the technological differences have not yet been sufficiently resolved to combine experimental results from different platforms [3,4]. Regardless of the overall interpretation, both sides hypothesize that some of the differences may be attributable to the presence of splice variants [5-9].

Although alternative splicing is a logical source of cross-platform differences, there has been no direct evidence to show that this is the case. Studies have indicated 40% or more of all human genes are alternatively spliced [10,11] and expression measurement differences may arise when probes on different platforms target differentially expressed splice variants of the same gene. It has been previously demonstrated that a sequence matching method between probes increases cross-platform consistency and reproducibility [12]. Others have matched probes to genes and shown that annotation discrepancies affect analysis [13]. Here, we combine the two ideas to provide evidence for alternative splicing based cross-platform disagreement.

We created an in-depth probe/genome/transcript annotation using the AceView transcript database. AceView is a comprehensive annotation of transcripts and genes that incorporates data from GenBank, dbEST and RefSeq [14]. It has been shown to offer a richer view of the transcriptome, with 3 to 5 times more high-quality transcript forms than UCSC known genes, RefSeq or Ensemble [14]. Capturing transcript diversity is important because probes may be derived from the same loci, but match different transcript sequences due to alternative splicing.

Using this new annotation, we categorized genes on each platform according to their susceptibility to splice variant differences and measured their cross-platform agreement in a biological data set using a traditional correlation measure and a Euclidean distance measure. The novel usage of the distance measure lends itself to a visualization that can show alternative splicing differences or other poorly performing probes.

## Results

### Matching platform-specific probes to AceView and RefSeq Transcripts

We created a transcript-level annotation of microarray probes to study the effects of alternative splicing on cross-platform microarray discordance. Microarray probe sequences from Affymetrix (U95Av.2 GeneChip, 25 mer oligonucleotide probes), Agilent (Human 1, cDNA probes) and Codelink (Uniset Human I Bioarrays, 30 mer oligonucleotide probes) were aligned to the genome and annotated as matching transcripts through shared genomic coordinates from the AceView and RefSeq [15] transcript databases (see Methods). Table 1 shows the results of both the genome and transcript mappings.

**Table 1:** Number of probes on each platform with AceView and RefSeq genome and transcript alignments

	Unique Probes	Genome Alignments	AceView Alignments	RefSeq Alignments
**Agilent**	13335	12676 (95%)	9698 (73%)	6901 (52%)
**Codelink**	9969	9855 (99%)	9330 (94%)	8243 (83%)
**Affymetrix**	199015	193006 (97%)	179740 (90%)	153611 (77%)

"Unique probes" is the number of probes on each platform that had sequences to be aligned. The other alignment categories indicate the number of probes that had a specific alignment.

Greater than 95% of all probes on the three platforms had genome alignments. Overall, 73%, 94% and 90% of Agilent, Codelink and Affymetrix probes, respectively, had AceView transcript alignments. The comparatively fewer alignments for Agilent stem from the strict coordinate restrictions we placed on the multiple-exon cDNA genome alignments. A detailed account of probe alignment conditions is available [see Additional file 1].

As anticipated, more probes were found to match to AceView transcripts than RefSeq transcripts, with 21%, 11% and 13% more total probes for Agilent, Codelink and Affymetrix, respectively. The rest of our analysis was therefore conducted using AceView data. Agilent and Codelink utilize single probes to target a gene and generate expression measurements. The Affymetrix U95Av2 chip is different. It targets a gene with a probe set consisting of up to 16 probes and summarizes the probe set to generate expression measurements. To create a transcript-level annotation of probe sets, a probe set was said to target a transcript if 5 or more probes matched it. An internal study showed five probes are necessary for reliable summarization measurements [see Additional file 2]. Of the 12453 Affymetrix probe sets, 11564 matched at least one transcript with 5 or more probes.

### Genes categorized by susceptibility to alternative splicing based differences

To detect the effects of splice variation on gene expression measurements, we categorized each gene by probe specificity against known splice variants as annotated in AceView. Genes most susceptible to expression measurement differences from splice variants are those in which the probes from each of two different platforms interrogate mutually exclusive, or disjoint, sets of transcripts. Genes that are not susceptible to these measurement differences are those in which probes on both platforms target the same, or equal, sets of transcripts. In between the two extremes are genes that are susceptible to splice variants, but the effect of which cannot be measured because the platforms target common transcripts as well as transcripts specific to each platform. Using our transcript annotation results, we categorized genes commonly targeted by each pairwise platform combination based on their susceptibility to alternative splicing. To match the Affymetrix gene expression data used below, the Affymetrix probe set annotation was used, as described above. The number of genes in each category for Affymetrix/Agilent, Affymetrix/Codelink and Agilent/Codelink is shown in Table 2.

**Table 2:** Pairwise platform gene classification based on susceptibility to alternative splicing

Platform A	Platform B	Common Genes	Equal A = B	Disjoint A ⋂ B = Ø	A\B ≠ Ø	B\A ≠ Ø
Affymetrix	Agilent	5804	1964	158	1599	3461
Affymetrix	Codelink	6429	2808	85	2526	2623
Agilent	Codelink	5183	1648	196	1450	3173

Let A be the set of transcripts a probe(set) on platform A targets

Let B be the set of transcripts a probe(set) on platform B targets

Equal A = B : Number of genes in which platform A and B target equal transcript sets.

Disjoint A ⋂ B = Ø: Number of genes in which platform A and B target disjoint transcript sets.

A\B ≠ Ø: Platform A and B target the same gene, but platform A targets extra transcripts that B does not.

B\A ≠ Ø: Platform A and B target the same gene, but platform B targets extra transcripts that A does not.

Note: The total number of classified genes can exceed the number of common genes because a gene may be a member of both A\B = Ø and B\A = Ø.

### Correlation measure of alternative splicing discordance

**Figure 1:**
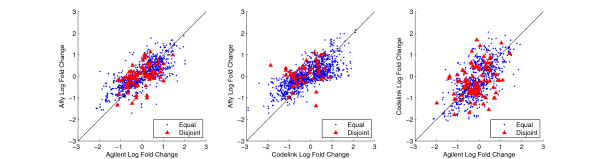
**Log2 fold change for equal and disjoint genes in each pairwise platform combination**. Each mark corresponds to a gene shared by two platforms. The diamonds are those genes that have equal transcript sets targeted by both platforms. The triangles are those genes that have disjoint transcript sets targeted by both platforms.

We looked for overall splice variant differences utilizing gene expression data from a previous biological experiment. RNA was obtained from PANC-1 cells of a pancreatic ductile cell phenotype and an early stage of their differentiation to a pancreatic islet phenotype (see Methods). Five technical and biological replicate microarray experiments were run on each platform and their results were averaged to produce a single fold change value for each gene. We analyzed the expression data by creating scatter plots of the log2 fold changes for genes in the equal and disjoint transcript sets for each pairwise platform combination, shown in Figure 1. Only genes that were statistically significant at p-value < 0.05 on at least one platform were included in our analysis. Table 3 shows the computed Pearson and Spearman correlation coefficients for each of the disjoint and equal gene groups.

**Table 3:** Pearson correlation coefficients for equal and disjoint gene groups

			Equal					
			Pearson			Spearman		

Platform A	Platform B	N	r	95% CI	P-value	r	95% CI	P-value

Affymetrix	Agilent	966	0.769	.742 < p < .794	<0.0001	0.783	.757 < p < .8058	<0.0001
Affymetrix	Codelink	1263	0.73	.703 < p < .755	<0.0001	0.745	.719 < p < .769	<0.0001
Agilent	Codelink	908	0.662	.624 < p < .697	<0.0001	0.652	.613 < p < .688	<0.0001

			Disjoint					

			Pearson			Spearman		

Platform A	Platform B	N	r	95% CI	P-value	r	95% CI	P-value

Affymetrix	Agilent	101	0.506	.344 < p < .638	<0.0001	0.53	.373 < p < .658	<0.0001
Affymetrix	Codelink	49	0.246	-0.03 < p < .493	0.0889	0.28	-0.0009 < p < 0.521	0.051
Agilent	Codelink	114	0.282	.104 < p < .443	0.0023	0.242	.061 < p < .408	0.0095

r – correlation coefficient

95% CI – rho is the 95% confidence interval around r calculated using Fisher's transform

p-value – value for testing the hypothesis of no correlation against the alternative that there is a non-zero correlation

n – number of genes in each pairing used in correlation calculation

There is a drastic drop in the correlation coefficients from the equal to the disjoint transcript sets for all three platform pairs; the difference being 0.263, 0.484 and 0.38 for Pearson and 0.253, 0.465, and 0.41 for Spearman from the Affymetrix/Agilent, Affymetrix/Codelink and Agilent/Codelink comparisons, respectively. As genes with disjoint transcript sets are most susceptible to alternative splicing based differences and genes with equal transcript sets are unsusceptible to alternative splicing based differences, the drop in the correlation coefficients between these two groups suggests alternative splicing is a contributing factor to platform discordance.

### Distance measure of alternative splicing discordance

**Figure 2:**
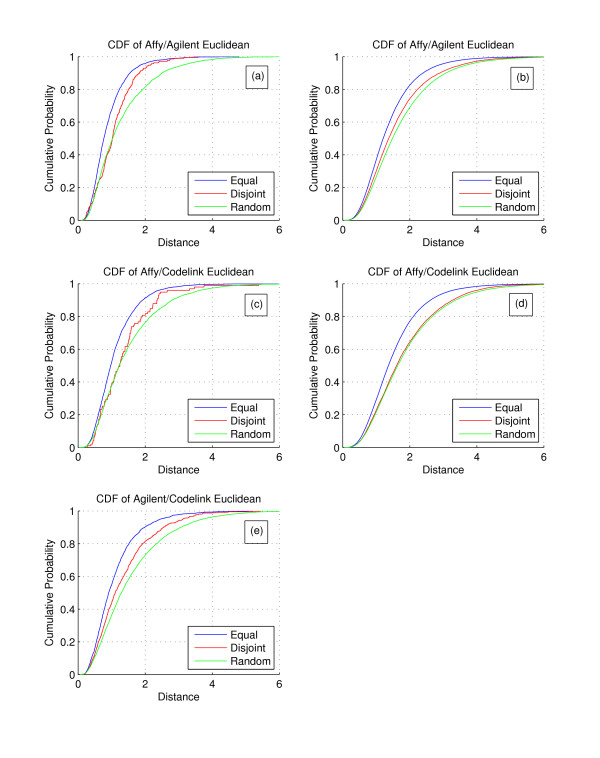
**CDFs of cross-platform distances**. a) CDF of gene-level distances for the Affymetrix/Agilent combination. b) CDF for probe-level distances for the Affymetrix/Agilent combination. c) CDF for gene-level distances for the Affymetrix/Codelink combination. d) CDF for probe-level distances for the Affymetrix/Codelink combination. e) CDF for probe-level distances for the Agilent/Codelink combination (probe-level after cross-hybridizing probes were removed). Genes with equal transcripts show higher agreement than genes with disjoint transcripts in terms of distance between log2 fold change expression vectors across platforms, as shown in 2a and 2c. The equal CDF rises faster and is farther to the left because a higher percentage of distances are small. Disjoint transcripts are more agreeable than random genes for the same reason, indicating transcript forms are regulated together better than random. Individual probe distances, shown in 2b, 2d and 2e, show the same pattern as the gene-level CDFs with equal, disjoint and random following each other in decreasing agreement. Affymetrix disjoint probes are not much improved over random, unlike the gene-level CDFs, indicating Affymetrix's reliance on redundant probe sets to obtain accurate expression data

A distance measure provides an alternative view of the data to confirm the correlation coefficient results. We calculated the log2 fold change of experimental versus control groups for each of the five replicates individually, creating a vector of the five fold change values for each gene on each platform. Using log2 fold change places each platform into a common measurement space. We then calculated the Euclidean distance between expression vectors from different platforms for the genes in the equal and disjoint transcript sets. Unlike the previous scatterplots, no restriction was made on statistical significance of the gene. Next, we plotted a cumulative distribution function (CDF) of all calculated distances for each grouping to highlight the differences attributable to alternative splicing, shown in Figure 2(a,c,e).

A curve that rises steeply and is shifted to the left represents a distance distribution that includes smaller distances than a curve shifted farther to the right. Smaller distances between expression vectors across platforms indicates higher agreement. The CDF curve for probes with equal transcript sets (impervious to alternative splicing based differences) is shifted to the left and rises faster than the CDF curve for disjoint transcripts sets (most susceptible to alternative splicing based differences) for all pairwise platform combinations. Thus, equal transcript sets tend to have smaller distances and higher agreement than disjoint transcript sets, indicating a distinct alternative splicing effect in platform discordance.

To establish a baseline for comparison, we randomly paired expression vectors from genes on different platforms and calculated the distances. A CDF of distances from unrelated expression vectors provides an unbiased worst-case distribution and the baseline CDF is plotted in Figure 2. By comparison, if two platforms agreed completely, the CDF would be a unit step function. Platform distance distributions based on equal and disjoint gene sets fall in between the two extremes, as the baseline CDF is shifted to the right, establishing the distance for unrelated measurements.

### Probe-level distance measure

Agilent and Codelink both use single probes to target a gene, but the Affymetrix U95Av2 genechip utilizes probe sets consisting of 16 probes to target a gene. Probe set gene expression measurements are statistical summarizations of member probes, which can be influenced by dead probes, cross-hybridization and other effects [16,17]. In order to generate a clearer view of expression measurements and agreement levels, we thought to analyze probe-level expression vectors instead of probe set summarizations for Affymetrix platform combinations.

We conducted a similar analysis as before, creating a vector of fold change values from the five replicates for each individual probe. To better isolate the effects of alternative splicing, we reduced the effect of cross-hybridization by removing from the analysis the 6.5%, 3.1% and 3.5% of individual Affymetrix, Agilent and Codelink probes, respectively, that matched multiple AceView gene symbols. Probe cross-hybridization results, as implied by sequence, are shown in Table 4.

**Table 4:** Percentage of probes cross-hybridizing to multiple AceView gene symbols

Platform	Number of Probes with AceView Alignments	Number of probes matching a unique AceView Gene Symbol	Percent probes matching multiple symbols
Affymetrix	179740	168001	6.53%
Agilent	9698	9397	3.10%
Codelink	9330	9000	3.54%

For all genes shared between platforms, individual probe-probe comparisons using the probe annotations were made for equal or disjoint transcript set targeting. By examining all possible combinations of individual probes, it was hoped that details masked by the probe set annotation would become apparent. We created CDFs of all of the probe distances in each category and established a baseline by randomly pairing probe expression vectors, shown in Figure 2(b,d). The alternative splicing effect is again apparent, with a left shift of probe distance CDFs for the equal transcript set versus disjoint transcript set.

### Kolmogorov-Smirnov Test

The Kolmogorov-Smirnov test is used to determine if two samples are drawn from the same underlying distribution. We used it to test whether the Equal-Disjoint, Equal-Random and Disjoint-Random CDF combinations for each of the platform pairs differ from each other. Table 5 illustrates the test results, laid out to match the ordering of the platform pairings in Figure 2. At p < 0.05, we reject the null hypothesis of drawing from the same distribution for all CDF combinations except for Disjoint-Random on Affymetrix probeset/Codelink (c). We accept that the Equal, Disjoint and Random distance distributions are different from each other, except in the case of Disjoint-Random for Affymetrix/Codelink (c), where we cannot reject the null hypothesis that they are the same.

**Table 5:** Kolmogoriv-Smirnov Test Statistics

Affymetrix/Agilent (a)			Affymetrix/Agilent (b)		
	p	K-S Stat		p	K-S Stat

Equal-Disjoint	<0.0001	0.2173	Equal-Disjoint	<0.0001	0.0953
Equal-Random	<0.0001	0.2118	Equal-Random	<0.0001	0.1464
Random-Disjoint	0.0033	0.1266	Random-Disjoint	<0.0001	0.0554

Affymetrix/Codelink (c)			Affymetrix/Codelink (d)		

	p	K-S Stat		p	K-S Stat

Equal-Disjoint	0.0025	0.1845	Equal-Disjoint	<0.0001	0.1272
Equal-Random	<0.0001	0.1598	Equal-Random	<0.0001	0.14
Random-Disjoint	0.3013	0.098	Random-Disjoint	0.0041	0.0188

Agilent/Codelink (e)					

	p	K-S Stat			

Equal-Disjoint	<0.0001	0.148			
Equal-Random	<0.0001	0.2173			
Random-Disjoint	0.0001	0.0941			

The distance distributions are different, indicating the shifts towards smaller distances and better agreement are meaningful in Figure 2. However, the disjoint transcript set in any pairwise combination involving Affymetrix probes (b, d) is not appreciably better than random, unlike the probe set-level CDFs, indicating Affymetrix's reliance on probe sets and robust algorithms for accurate expression measurements.

### Individual gene expression visualization

**Figure 3:**
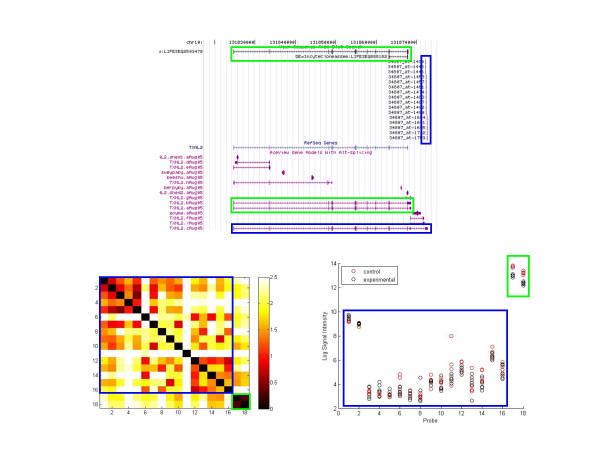
**Visualization of TXNL2**. Top: UCSC Genome Browser view of TXNL2 probe alignments alongside AceView transcripts. The probes and transcripts they target are outlined by color; Agilent is green, Affymetrix is blue. Bottom Left: Heatmap visualization of the gene according to pairwise Euclidean distance. The Agilent probes, outlined in green, form a dark block, indicating their measurements are close together. The Affymetrix probes do not show any grouping pattern. Bottom Right: Log2 signal intensity of the replicates. The Affymetrix probes, outlined in blue, show low signal intensity and a random distribution of control and experimental signals from probe to probe, which explains the lack of visual pattern in the heatmap. The Agilent probes, outlined in green, show clear separation between the control and experimental groups, indicating a downregulation of TXNL2. Real-time PCR showed the Affymetrix transcript to be absent in the sample, while the Agilent transcripts were validated to be downregulated, explaining the signal patterns.

**Figure 4:**
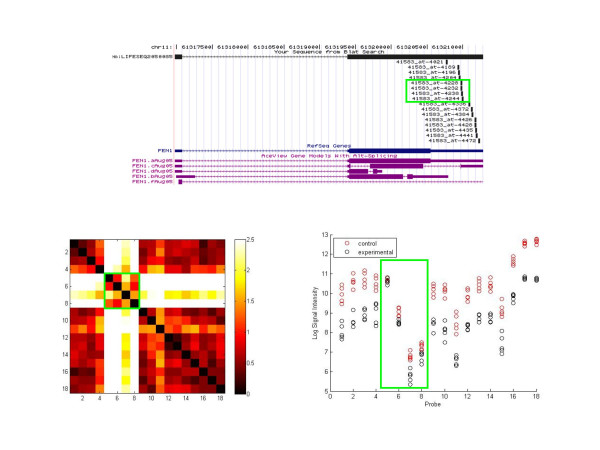
**Visualization of FEN1**. Top: UCSC Genome Browser view of FEN1 probe alignments alongside AceView transcripts. The four misbehaving probes are outlined in green. Bottom Left: Heatmap visualization of the genes according to pairwise Euclidean distance. Probes 1–16 are Affymetrix, 17 is Agilent, and 18 is Codelink. Probes 5–8 (green outline) are distant from the rest of the probes in the set, creating a white cross in the symmetric matrix. The rest of the probes are all in high agreement with each other with short distances between expression vectors. Bottom Right: Log2 signal intensity of the replicates. The signal intensities of the misbehaving probes, outlined in green, do not show the same high degree of separability between the control and treatment groups that is exhibited by the rest of the probes. The signal intensity graph confirms that these probes behave differently and should form their own cluster, as found in the heatmap.

We generated a distance matrix containing all pairwise probe distances for each gene. By turning the matrix into a heatmap colored by distance, with probes ordered by their position along the transcript, clusters of probes with signals showing high agreement and similar patterns show up as darker blocks along the main diagonal. Figures 3 and 4 are examples of the visualization for the genes thioredoxin-like 2 (TXNL2) and flap structure-specific endonuclease 1 (FEN1), respectively. TXNL2, otherwise known as PICOT (protein kinase C interacting cousin of thioredoxin) is a hypertrophy-inducible, PKC-inhibiting negative regulator of hypertrophy. No biological function has as yet been ascribed to be splice-variant specific. FEN1 removes 5' overhanging flaps in DNA repair and processes the 5' ends of Okazaki fragments in lagging strand DNA synthesis. The top part of each figure shows the AceView transcript forms and where the probes align.

TXNL2 is a case of disjoint transcript sets being targeted by Affymetrix and Agilent. The Affymetrix probe set targets TXNL2.cAug05 (outlined in blue). Two Agilent probes target TXNL2.bAug05 (outlined in green). In the heatmap, the Agilent probes clearly form a dark cluster distinct and distant from the Affymetrix probes. Examining the log2 signal intensities, Agilent shows a clear separation between the control and experimental groups while Affymetrix shows no pattern at all. Agilent microarray results indicate that TXNL2 is downregulated, with fold changes of -1.78 (p = 0.003) and -1.67 (p = 0.007). Affymetrix microarray results indicate no change, with a fold change of -1.04 (p = 0.632).

These results were validated by PCR experiments. Real-time PCR showed a fold change of -1.70 (p = 0.014) for the transcripts targeted by Agilent. The transcript targeted by Affymetrix failed to amplify after 40 cycles. The lack of amplification corroborates the low signal intensity and lack of signal pattern in the microarray data, meaning the Affymetrix transcript is likely not present in the sample. Therefore TXNL2 represents a gene whose discordant expression measurements are attributable to alternative splicing, because each platform targets mutually exclusive transcripts with different, validated results. There were three additional transcripts for which we could adequately design a real-time PCR assay and for which probe alignments are shown [see Additional file 3]. In the first case, CDC42 was significantly downregulated when assayed by Codelink (-1.65, p = 0.00298; variants e, f) but unchanged by Affymetrix (1.10, p = 0.04; variant d). Surprisingly, we were not able to validate either result by real-time PCR (variants e, f: 1.82, p = 0.07; variant d: 2.97, p = 0.02), suggesting a false positive result for Codelink and a false negative one for Affymetrix. In two other cases where increased transcript expression was detected by Affymetrix (CALM1 (variant b), 1.94, p < 0.003) and by Codelink (FMO5 (variants a, d), (2.23 p < 0.001), both were validated by real-time PCR (2.73, p = 0.003; 3.01, p < 0.001). Correspondingly, results from the Agilent platform showed no change for different variants of both genes, (1.11, p = 0.45; -1.26, p = 0.59 for CALM1 (variant a) and FMO5 (variant c), respectively). Nevertheless, we further determined by real-time PCR that these specific variants were also upregulated (8.06, p < 0.001; 2.69, p < 0.001). These results demonstrate that additional factors clearly contribute to the dissociation of results across platforms, and that splice variants may only play a partial role in explaining these differences.

One of these factors might be the existence of poorly performing probes possibly resulting from failure to hybridize, or susceptibility to cross-hybridization. FEN1 is an example of a visualization revealing poorly performing probes. A group of four Affymetrix probes, outlined in green, are distant from all of the other probes for the gene, including the Agilent and Codelink probes (probes 17 and 18 respectively). Due to an existing non-disclosure agreement with GE Healthcare, we are unable to show the position of the Codelink probe on the genome, but it is in the vicinity of Affymetrix probe 41583_at-4021. The group of four probes shows poor or no separation in the log2 signal intensity between the control and experimental groups, unlike the other probes that show clear downregulation. This difference is not due to transcript specificity and highlights the fact that individual probes can misbehave for other reasons.

## Discussion

Mapping probes to transcripts via the genome yields more information and is more complex than a direct probe/transcript mapping. Genome coordinates show the cause of alignment failures, such as probes aligning to an intron. In addition, genome builds are more stable than transcript databases and allow for enhanced future study. For instance, a probe may have been designed using proprietary transcript sequences or map to an unexplored area where tiling arrays indicate transcriptional activity [18,19]. While we may miss some alignments involving rare non-standard introns or some Agilent cDNA clones due to our algorithm, we maintain a high stringency of matches between genome coordinates, probes, and transcripts.

The algorithms that generate genomic alignments and transcript annotations differ for the three platforms. They serve as an abstraction layer to transform the different probe designs from each platform into commonly comparable annotation data. Annotation quality is the key factor when investigating alternative splicing disagreement. Although we maintain a high stringency in the alignments, the Agilent cDNA probes have lower transcript hybridization specificity than Affymetrix or Codelink due to their length. Therefore, under actual hybridization conditions, Agilent may target more transcripts than our purely sequence-based annotation indicates. While this may slightly impact the Agilent results, our annotation provides a good starting point for alternative splicing analysis. For instance in TXNL2, Agilent and Affymetrix target disjoint transcripts based on sequence alone. It is possible that Agilent could bind to the Affymetrix transcript, TXNL2.cAug05, though we do not know for sure. Even so, we were able to assay the terminal exon exclusive to Affymetrix to show splicing-based disagreement. Screening for such candidate genes most susceptible to alternative splicing requires at least a detailed sequence-based annotation.

The drop in correlation coefficients of expression measurements across platforms from genes impervious to alternative splicing based differences to those genes most susceptible to alternative splicing based differences seems to indicate alternative splicing plays a role in platform discordance. Although correlation coefficients are widely used measures of platform concordance, the large drop was not necessarily supported by a corresponding visual change in the distribution of data points underlying the coefficient calculations.

We verified the correlation coefficients by using a distance measure derived from gene and probe-level log2 fold change data from replicate samples. Instead of using other fuzzy measures such as percent gene list overlap to measure improvement, our distance measure provides a clearer measure that cuts to the signal differences attributable to alternative splicing. The CDF shows that probes that target equal transcripts have fold changes that on average are closer and more agreeable between the two platforms than probes with disjoint transcripts. A key point is that the CDFs were generated without any arbitrary p-value or fold change filtering of the probes. Since we are not concerned with methods to improve concordance, we did not utilize any filtering method or thresholding that could change the interpretation of microarray results. The CDF differences show that alternative splicing is a layer on top of the basic platform technology differences that contributes to platform discordance, independent of any post-processing method.

While alternative splicing clearly affects platform discordance, making a broad-based quantification of its effects is difficult. First, alternative splicing is tissue dependent [20]; not only do the variants need to be differentially expressed, but the genes affected will change in each experiment. While we categorized genes based on their susceptibility to alternative splicing differences, the majority of genes have both common and specific transcript targets on each platform, making it difficult to isolate the effect of alternative splicing from the underlying technological discordance represented in the common transcripts. Though it is not possible to establish a universal boundary on alternative splicing's contribution to discordance in this study, we have provided evidence for its existence.

Basic platform technology differences are revealed in the CDFs for genes with equal transcript sets on any two platforms compared. These genes are unsusceptible to alternative splicing differences, yet the CDF does not approximate a stair-step function that would indicate complete agreement. Other platform differences may include high probe/mRNA secondary structure, difficulties in labelling and other design and protocol considerations. Therefore underlying platform-based differences exist and are unexplainable by alternative splicing alone. In this respect, our inability to validate some of our discordant microarray results demonstrates that clearly other factors exist. To aid in understanding this phenomenon, our distance measure also offers a way to visually examine similarly performing probes. In a heatmap of pairwise probe distances for a gene, clusters of probes became visually apparent based on shared signal intensity patterns according to positional ordering. The presence of clusters of "close" probes provides both insights into probe performance behaviour and a sanity check on the distance measure itself. However a more in-depth study using other clustering techniques on different factors could show probes grouped together on the basis of GC content or hybridization conditions, revealing the causes of differing probe behaviour for the same transcripts.

In addition to probe design-dependent differences in sensitivity and specificity, systematic and random sources of variability unrelated to probe sequence can contribute to an "intrinsic" level of discordance across expression arrays. For instance, context-dependent differences in expression data have been reported across generations of Affymetrix arrays with identical probe sequences [21]. Moreover, a study by the MAQC consortium showed that cross-site concordance rates for a single array platform was ~50–65% for the most highly ranked differentially expressed genes when two related samples were compared [[22], Fig. S2]. This within-platform agreement rate is noteworthy compared to the ~40% cross-platform agreement rate previously reported [6,9,23]. In our current analysis, more detailed ANOVA models were used to describe and subsequently remove non-biological sources of variability specific to each platform, such as batch effects due to RNA extraction and array- and dye-specific bias. Assuming that systematic errors typical of a microarray experiment can be accounted for in this manner, the remaining discordance is still only partly explained by the presence of splice variants. In this regard, a similar re-analysis of other multi-platform datasets would be highly interesting and our code is made available for this purpose [see Additional file 4].

As known transcript diversity increases, we expect the number of genes with equal transcripts on both platforms to decrease, raising the confidence of the annotation of the remaining genes, producing lower distances and a sharper CDF. Despite the certain existence of technical variables affecting cross-platform concordance, we believe inter-platform agreement rates can be further improved as use of the transcriptome for probe annotation increases. To further advance this goal, we have recently provided custom chip-description-files and annotation files for a transcript-based probe set redefinition of the human Affymetrix arrays [24].

## Conclusion

Using an in-depth transcript annotation, we categorized genes as being either impervious or susceptible to alternative splicing cross-platform differences. Examining gene expression data for these genes in two manners, we provided evidence that alternative splicing cross-platform discordance exists. Utilizing a distance measure, we were able to create a gene visualization that can highlight probe differences. Alternative splicing's contribution depends heavily on the prevalence of alternative splicing in the experimental data set and it is a factor adding to omnipresent and underlying technical differences.

## Methods

### Generating probe genome coordinates

All alignment code was run on the NIH Biowulf supercomputing cluster. Probes were aligned to Human Genome Build (hg17, May 2004). Although a new genome build was released in March 2006, the AceView transcript genome coordinates have not yet been updated to reflect the new build.

### Agilent

Agilent probe sequences were generously contributed by Open Biosystems (Incyte clone database). Of the 13323 unique Agilent cloneIDs, sequences were available for 12955 clones. Of the 12955 clones, 10016 were annotated as being a confirmed full length cDNA clone sequence; the other 2939 were potential full length clones with a 5' read. Of the 2939 potential full length probes, 12 had additional 3' read sequences. Potential full length probes may have gaps that are not sequenced.

The Agilent probes were aligned to the genome using the default parameters of the BLAT algorithm. Alignment results were filtered using the following steps:

1. For each probe, the ratio between the number of matching base pairs in the alignment and the number of base pairs in the probe sequence had to be greater than 0.75 for the highest scoring alignment. This requirement forces the best alignment to match a majority of the probe sequence, since BLAT produces many spurious short alignments with 100% percent identity.

2. For each probe, accept any alignment whose score is within 5% of the best alignment's score. This requirement eliminates low quality alignments, while allowing for finding coordinates of gene repeats.

#### Affymetrix and Codelink

Affymetrix probe sequences for the U95A.v2 chip were downloaded from the Affymetrix website [25], yielding sequences for 199015 unique probes after removing 69 probes associated with ALU repeats. Codelink probe sequences for 9969 unique probes were obtained from GE Healthcare.

Traditional sequence alignment algorithms, such as BLAST are unsuited for aligning short probe sequences to the genome because they work best with sequences greater than 50 bp and require parameter tuning and post-processing quality control for optimal results. Other programs that allow for fuzzy alignments to mimic hybridization conditions, such as GCG's *findpatterns *or EMBOSS's *fuzznuc *do not scale well with 200000 probes at the genome level. We therefore created a custom alignment procedure, written in perl. It is a cascade architecture that progressively uses more computationally intensive alignment algorithms on the reduced subset of probes unaligned in the previous step, making efficient use of computational resources. The algorithm consists of four steps:

1. Exact alignments of probe sequences to the genome are found using a hash table. A sliding window is passed over the genome, querying the "dictionary" hash table for a probe match using the window sequence as the key.

2. Probes without exact matches are then tested for alignment with single base pair mismatches, again using a hash table approach with an expanded dictionary to include all possible mismatch sequences. When windowing the genome, a "don't care" term corresponding to an N nucleotide are substituted at each position in the window when querying the hash table, effectively scanning the genome n times, where n is the probe length.

3. Probes not aligned in either step above are subjected to regular expression genome searches to determine whether a probe spans an intron. To aid in intron search specificity, intron feet are added to each regular expression because 99% of all introns begin and end with the sequence GT-AG or GC-AG. Only those probes found with intron gaps of less than 60 kbp are accepted to eliminate spurious alignments. Regular expressions are slow compared to hash-based approaches.

4. Any remaining probes are run through BLAT as a catch all. A previous study (GE, personal communiqué) shows that probes can tolerate up to 3 mismatches with the target sequence before there is significant signal degradation due to failed hybridization. A conservative BLAT score cutoff is used, equating to accepting either two mismatches or a gapped intron alignment and one mismatch. BLAT is only guaranteed to find perfect matches down to 33 bases and therefore may miss some probes of interest due to short probe length.

### Matching probes to transcripts

Two transcript databases, AceView and Reference Sequence (RefSeq) were used. AceView human Aug05 transcripts and genome coordinates were obtained from the AceView website [26]. Genome coordinates of RefSeq transcripts as determined by BLAT alignment were obtained from UCSC on 12/29/05. Individual probes were then matched to transcripts by comparing common genome coordinates in an Oracle database query.

### Experimental data

Expression data for all three platforms were obtained as described in [9]. PANC-1 cells were grown in serum-rich medium, trypsinized and collected immediately and at 24 h following transfer of these cells to serum-free medium. Each one of the microarray platforms utilized a common sample pool of RNA from control PANC-1 cells which have a pancreatic ductal cell phenotype or from an early stage of their differentiation to a pancreatic islet phenotype. RNA was labeled and hybridized to microarrays from Affymetrix (U95Av.2 GeneChips, multiple 25 mer oligonucleotide probe sets), Agilent (Human 1, cDNA probes) and Amersham (Codelink UniSet Human I Bioarrays, 30 mer oligonucleotide probes) according to manufacturers' guidelines. For each time point, three arrays were hybridized with RNA derived from one of the PANC-1 cell cultures (technical replicates); the remaining two microarrays were hybridized with RNA from two independent cell cultures (biological replicates), thus generating five data points for each probe at each time point.

Raw data from Codelink was median-normalized as recommended by the manufacturer. Raw data from the Agilent platform underwent lowess normalization via the Feature Extraction Software. Affymetrix gene expression measurements underwent quantile normalization and summarization using RMA [27]. Experiment-wide systematic technical or "batch" effects such as that caused by RNA extraction on different sample batches were removed using Partek Genomics Suite [28].

For Codelink and Affymetrix GeneChip data, the following ANOVA model was used: let Y_gij _be the base-2 logarithm of the background-corrected measurement from gene g (g = 1...n), treatment i (i = 1,..2), and RNA extraction batch j (j = 1,..2):

Y_gij _= μ + T_i _+ R_j _+ (TR)_ij _+ ε

where T is the main effect for treatments, R is the main effect for RNA extraction, and TR is the interaction effect of RNA extraction and treatment, and ε is stochastic error. Using this model, we were able to subtract the effect of RNA extraction from the gene expression signal.

For the Agilent 2-channel array, the following ANOVA model was used: let Y_gij _be the base-2 logarithm of the background-corrected measurement from gene g (g = 1,...n), treatment i (i = 1,2), RNA extraction batch j (j = 1,2), configuration on slide k (k = 1,2), dye l (l = 1,2) and array m (m = 1,...5).

Y_gij _= μ + T_i _+ R_j _+ (TR)_ij _+ (C)_k _+ (D)_l _+ (AR)_jm _+ ε

where T is the main effect for treatments, R is the main effect for RNA extraction, and TR is the interaction effect of RNA extraction and treatment, C and D are the effects for configuration and dye respectively, and AR is the interaction effect of RNA extraction and array and ε is stochastic error. Effects of RNA extraction, slide configuration (left vs. right), dye, and array were removed. Cy5 and Cy3 channel signals were then averaged for each sample. All raw and processed data are available in GEO (GSE7785).

### Real-time PCR Expression Analysis

An inventoried assay for the gene TXNL2 was obtained from Applied Biosystems (Hs01582641_g1) and was used to test for the TXNL2.aAug05 and TXNL2.bAug05 transcripts targeted by the Agilent probes. The assay's context sequence is TGGATATTGTGAAGGAACTGAAAGA.

Custom designed PCR primers were synthesized by MWG-BIOTECH Inc. (High Point, NC, USA). The forward (CCCAAAGTGCTGGAATTTACAGGAGTGT) and reverse (TGGTGAATGAGGCATCAGGAAGCTA) primers were designed to target the same exon as the Affymetrix probe set and align to TXNL2.cAug05. Melting point Tm temperatures (65.9°C and 64.8°C respectively) and %GC content (46.4% and 48.0% respectively) was checked using Primer Express™ software package from Applied Biosystems (Foster City, CA, USA).

### Analysis

Equal and disjoint transcript sets for probes and genes were determined using Access database queries examining the transcript annotation data. The correlation, distance measure and CDF analysis was conducted using built-in Matlab functions, custom Matlab scripts, custom Perl scripts and Microsoft Access databases to tie the data together. Code and data are available as supplementary files.

## Authors' contributions

JCL developed the methods, carried out the analysis and drafted the manuscript. JCL and MCC designed the study. DS performed the real-time PCR experiments. JL and DS assisted with the figures. MCC conceived of the study. All authors read and approved the final manuscript.

## Supplementary Material

Additional file 1 Probe alignment failure conditions. Provides examples of probes failing to align for various causes. Click here for file

Additional file 2 Minimum number of probes for reliable Affymetrix summarization measurements. Graph showing the correlation coefficient versus number of probes in a probe set matching to RefSeq in a cross platform comparison between Affymetrix and Agilent. Click here for file

Additional file 3 Alignments and real-time PCR designs for 3 other "discordant" genes: CDC42, CALM1, FMO5. Alignments on the UCSC Genome browser for each platform's probe(set) against relevant AceView transcripts. Real-time PCR primers and probes are included. Click here for file

Additional file 4 Perl and Matlab code used for sequence matching and analysis. The readme file contains descriptions for each script. Click here for file
