# Supplementary material for: A detailed transcript-level probe annotation reveals alternative splicing based microarray platform differences
Source: BMC Genomics. 2007 Aug 20;8:284. doi: 10.1186/1471-2164-8-284 (PMC2000902; doi:10.1186/1471-2164-8-284)
Supplement: Additional file 1 — Probe alignment failure conditions. Provides examples of probes failing to align for various causes. [file 1471-2164-8-284-S1.doc]

Agilent cDNA clones showed the lowest alignment rate owing to the difficulty of finding genomic mRNA/DNA alignments. Agilent probes failed to align because they did not meet a 75% similarity match score requirement (probes 1958933, 5546493), contained poly-A tails (probes 3138407, 5038177) or aligned to floating contigs not present in our BLAT mirror (1593561). The Affymetrix and Codelink probes that failed to align had too many mismatches, insertions, or deletions to match hybridization conditions, or simply did not have a genome alignment, perhaps because they were from other species.

Although more probes align to AceView than RefSeq, there is still a significant decrease from the number of probe/genome alignments to the number of probe/transcript alignments for each platform. The decrease occurs for a number of reasons.

- *Probe aligns to an intron*

Agilent: 123530, 125844

Codelink: GE52943, GE52945, GE63124, GE63383

- *Probe aligns to no transcript, but is supported by a GenBank mRNA*

Codelink: GE52944

Affy: 1076_at-2033

- *Probe does not align to any known transcripts*

Agilent: 113553, 123312

Affy: 1129_at-364, 1009_at-211, 1009_at-223

- *Probe does not align because of our intron detection algorithm –*

Affy: 1016_s_at-808. The predicted exon’s location based on sequence matching is 114067639-114067642 on ChrX, whereas it actually is 114068186-114068190.

Our intron algorithm searches for the shortest possible gap between exons, but in some cases the next exon start is further downstream than predicted by sequence alignment.
