# Supplementary material for: A detailed transcript-level probe annotation reveals alternative splicing based microarray platform differences
Source: BMC Genomics. 2007 Aug 20;8:284. doi: 10.1186/1471-2164-8-284 (PMC2000902; doi:10.1186/1471-2164-8-284)
Supplement: Additional file 2 — Minimum number of probes for reliable Affymetrix summarization measurements. Graph showing the correlation coefficient versus number of probes in a probe set matching to RefSeq in a cross platform comparison between Affymetrix and Agilent. [file 1471-2164-8-284-S2.doc]

This figure plots the correlation coefficients of the log ratios between Affymetrix and Agilent gene expression data versus the number of probes in an Affymetrix probe set that align to a RefSeq transcript. If an Agilent probe and an Affymetrix probe set target the same gene, then the number of Affymetrix probes aligning to that gene’s RefSeq was found. Each data point represents the correlation of the log ratios for the genes in each RefSeq alignment bin. The gene expression data is from the same experiment as described in the main paper. Five probes aligning to a RefSeq is the minimum number of probes necessary for reliable statistical summarization of a probe set because it represents the beginning of the concordance plateau with a correlation of 0.6.
