# Supplementary material for: A detailed transcript-level probe annotation reveals alternative splicing based microarray platform differences
Source: BMC Genomics. 2007 Aug 20;8:284. doi: 10.1186/1471-2164-8-284 (PMC2000902; doi:10.1186/1471-2164-8-284)
Supplement: Additional file 3 — Alignments and real-time PCR designs for 3 other "discordant" genes: CDC42, CALM1, FMO5. Alignments on the UCSC Genome browser for each platform's probe(set) against relevant AceView transcripts. Real-time PCR primers and probes are included. [file 1471-2164-8-284-S3.doc]

**CDC42**

Codelink (GE57603): CDC42.eAug05, CDC42.fAug05

**Real-time PCR design:**

Forward: GTAATCTTTCCCACCTTCCCAAA

Reverse: GAGACATGAGAAAGCATTGGTTCA

Probe: TTCTTGTAGATGCATTAGTG

Affymetrix (39736_at): CDC42.dAug05

**Real-time PCR design:**

Forward: TGCTTGAGTTGCCTGATGCT

Reverse: AGACCAAGTTCCCTTTTGCAATAG

Probe: AGAGCTTTTTGGTTTGGATT

**CALM1**

Agilent: (5265655|CL=LIFESEQ5265655): CALM1.aAug05

**Real-time PCR design:**

Forward: AGCCTGTTAATCCAACCCAATG

Reverse: TGCTCCAGGCCCTCGAA

Probe: TGTAACGCCAGTTTGG

Affymetrix (41288_at): CALM1.bAug05

**Real-time PCR design:**

Forward: GTTGAGCGAGGCAAATGGAT

Reverse: TCCTTGGCAACAGTGCATCA

Probe: TCGATATTTCAGATGGGC

**FMO5**

Agilent (4759753|CL=LIFESEQ4759753): FMO5.cAug05

**Real-time PCR design:**

Forward: GGCAACAGCAGCTGAAATATAGAG

Reverse: GGGCTGAAGCTGCTTTGG

Probe: TTGTAGGATTAACTTTTTCG

Codelink (GE62762): FMO5.aAug05, FMO5.dAug05

**Real-time PCR design:**

Forward: CCTGTTTTCCATTTGTATTAACTCATCT

Reverse: GGGATTACCACAAGGAAGAGTGA

Probe: CTTCCACTCATGATCC
