# Supplementary material for: A detailed transcript-level probe annotation reveals alternative splicing based microarray platform differences
Source: BMC Genomics. 2007 Aug 20;8:284. doi: 10.1186/1471-2164-8-284 (PMC2000902; doi:10.1186/1471-2164-8-284)
Supplement: Additional file 4 — Perl and Matlab code used for sequence matching and analysis. The readme file contains descriptions for each script. [file 1471-2164-8-284-S4.zip › code/readme.rtf]

Preformatting:- affyformatter.pl - converts probe file into FASTA format and removes alu repeats- codelinkformatter.pl - converts probe file into FASTA format- agilent does not need a FASTA converter as clone sequences were provided in FASTA format- removeDups.pl - removes duplicate probes (by name) from a FASTA file- genomeformatter.pl - strips the newline characters from the chromosome builds so a line read reads an entire chromosomeProbe/Genome Matching:- findExactMatches.pl - finds exact alignments of probe sequences to the genome using a hash table approach (used for affy and codelink)- findOneSubMatches2.pl - finds alignments of probe sequences allowing for single base pair mismatches by hashing the genome with a wildcard character at every position in the probe.- findGappedExactMatches2.pl - finds alignments of probe sequences using regular expressions to search for probes aligning across intronic gaps- saveNonExact.pl, saveOneSubMatches2.pl, saveNonGappedExact2.pl, saveNonBlat.pl - each of these files takes the output of an alignment stage, finds which probes were not aligned, and then saves those probes in a smaller file for processing in the next stepPostformatting for database:- blatformatter.pl - reshapes the blat psl format file, putting each exon on its own line with its own coordinates- exactformatter.pl, onesubformatter.pl, gappedformatter.pl - takes output of the genome matching programs and shapes it into a delimited file- acemblyformatter.pl - takes the aceview transcript coordinate data and parses for exons/coordinatesAnalysis:- genesets.pl - database queries for to determine equal and disjoint transcript sets- getGeneIntensityValsCrossHyb.pl - links probe ids of interest to gene expression data stored in database- lowerBound.m, lowerBound_func.m - performs the random permutation of probes to generate a distance distribution of comparing independent probes- genevis.m, genevis_func.m - saves the gene expression data for each gene's probes in a separate file- geneFinalStats.m, geneFinalStats_func.m, getDistances.m - tabulates all of the distance dataFigures:figure1.m - draws correlation graphs shown in figure 1 of the paperfigure2.m - draws figure 2, a, c, and efigure3.m - draws figure 2 b and dfigure4.m - for figures 3 and 4, draws the heatmapfigure4_bottom.m - for figures 3 and 4, draws the log signal intensity plots of the probes
